# The benefits of radioactive iodine ablation for patients with intermediate-risk papillary thyroid cancer

**DOI:** 10.1371/journal.pone.0234843

**Published:** 2020-06-15

**Authors:** Xiaofei Wang, Jingqiang Zhu, Zhihui Li, Tao Wei

**Affiliations:** Department of Thyroid & Parathyroid Surgery, West China Hospital, Sichuan University, Chengdu, P.R. China; Chang Gung Memorial Hospital at Linkou, TAIWAN

## Abstract

**Background:**

The beneficial effects of radioactive iodine (RAI) ablation for intermediate-risk papillary thyroid cancer (PTC) patients are still controversial.

**Materials and methods:**

To determine the impact of RAI therapy on disease-specific survival (DSS) in patients with intermediate-risk PTC, we retrospectively analyzed the data of 23107 intermediate-risk PTC patients who underwent primary thyroidectomy with or without RAI in the Surveillance, Epidemiology, and End Results (SEER) database.

**Results:**

RAI therapy was significantly associated with improved DSS (adjusted HR = 0.65, *P* = 0.017) in intermediate-risk PTC patients after multivariate adjusting for clinicopathological characteristics. However, subgroup analyses demonstrated that RAI ablation was only associated with improved DSS in patients with male gender (adjusted HR = 0.47, *P* = 0.005), age ≥ 45 years (adjusted HR = 0.34, *P* < 0.001) and tumor size > 20 mm (adjusted HR = 0.58, *P* = 0.007).

**Conclusion:**

RAI decision-making should be considered on an individual basis rather than “one size fits all” in intermediate-risk PTC patients; only patients with male gender, age ≥ 45 years, and tumor size > 20 mm may benefit from RAI therapy.

## Introduction

Papillary thyroid cancer (PTC), accounting for 85% to 90% of all thyroid cancers, is the most common endocrine malignancy, and its incidence has sharply increased worldwide in recently decades [1,2]. Although PTC is generally considered to be an indolent tumor because of its excellent overall prognosis, approximately 30% of patients present recurrent or persistent disease, which is related to higher risk of disease-specific death [3,4].

Radioactive iodine (RAI) treatment has been considered as a highly accurate and targeted therapy with limited side effects for PTC because of the affinity of the thyroid for iodine. RAI after total thyroidectomy may ablate normal thyroid remnants and destroy suspected micrometastases or known persistent disease, which might improve disease-specific and disease-free survival. Early studies showed that RAI can improve the prognosis of patients with PTC [5–7]. For these reasons, RAI later became widely used in the treatment of patients with PTC [8,9]. Although the side effects of RAI are considered minimal, they do occur. Ample data has shown that RAI may cause some acute and long-term side effects [10–12], including nausea and vomiting, swelling and pain in the salivary gland, loss of taste, pulmonary fibrosis, second primary malignancies, and so on. Therefore, with the deeper understanding of the risks and benefits of this therapy, the adoption of RAI in the treatment of PTC has gradually become relatively conservative in recent years. According to the current American Thyroid Association (ATA) guidelines, high-risk patients should be routinely treated with RAI therapy after surgery, while low-risk patients should not be used. However, whether intermediate-risk patients should be treated with RAI therapy has been controversial, because there is no clear evidence that RAI will benefit their postoperative recurrence and survival.

A large number of retrospective studies have shown that RAI treatment may reduce the recurrence and disease-specific mortality in patients with intermediate-risk PTC [13–15]. Moreover, a recent retrospective study based on data from the National Cancer Database (NCDB) indicated that RAI therapy was associated with improved overall survival (OS) in patients with intermediate-risk PTC [16]. Unfortunately, this study did not analyze disease-specific survival (DSS), which might be more valuable than overall survival, because the overall survival might be affected by age and comorbidities. In contrast, other investigations did not show any benefit of RAI therapy, including a recent large-sample report from a single center in which RAI therapy did not reduce the risk of loco-regional recurrence in patients with intermediate-risk PTC, even in patients with aggressive characteristics such as BRAF positivity, multifocality, extrathyroidal extension, and regional lymph node metastases [17].

To further evaluate the benefits of RAI treatment, we conducted a retrospective analysis based on data from the National Cancer Institute Surveillance, Epidemiology, and End Results (SEER) program to investigate the effects of RAI therapy on disease-specific survival (DSS) among patients with intermediate-risk PTC. Furthermore, we tried to determine whether there was an association between RAI therapy and improved survival in a cohort of patients with specific risk factors.

## Materials and methods

### Data

A retrospective cohort study was performed using the data from the SEER database, which is a U.S. population-based cancer registry. This database represents approximately 28% of the U.S. population, capturing various information on patient demographic, clinical, tumor and treatment. The SEER program statistical analysis software package (SEER*Stat 8.3.5, available at https://seer.cancer.gov/seerstat/) was used to identify patients.

### Inclusion and exclusion criteria

Adult patients (≥ 18 years of age) diagnosed with PTC between 2004–2014 who underwent total thyroidectomy (TT), near total thyroidectomy (NTT) or subtotal thyroidectomy (STT) were identified using the following International Classification of Diseases for Oncology, Third Edition codes (ICD-O-3): 8050/3, 8260/3, 8340/3, 8341/3, 8342/3, and 8343/3. Our cohort was further restricted to patients with intermediate-risk PTC, defined as patients with microscopic extrathyroidal extension and/or with metastatic cervical lymph nodes (T3N0M0; T1-3N1M0) based on ATA risk criteria and American Joint Commission on Cancer (AJCC) staging.

Patients with aggressive variants of PTC, such as tall cell, columnar, sclerosing and insular variants, and poorly differentiated or anaplastic thyroid cancers were excluded. Patients with more than one primary site of malignancy were excluded. Patients with missing data on tumor size, extension, lymph node and distant metastases were excluded. Patients received external radiation treatment were also excluded. The study was granted exemption by our institutional review board.

### Study variables

Demographic variables included patient age at diagnosis, gender, race, and year of diagnosis. Age at diagnosis was divided four groups: < 45 years, 45–55 years, 55–65 years and ≥ 65 years. Pathologic and clinical characteristics included tumor size, extent of surgery (TT or NTT/STT), bilaterality (yes or no), multifocality (yes or no), extrathyroidal extension (yes or no), status of lymph node metastases (negative, positive or not examined), RAI therapy (yes or no), follow-up time, vital status (alive or dead), and cause of death. Tumor size was divided four groups: ≤10mm, 10–20 mm, 20–40 mm, and > 40 mm. The primary endpoint was disease-specific survival (DSS), which was defined as the time from diagnosis to the date of death caused by thyroid cancer or last follow-up. Deaths due to causes other than thyroid cancer were censored when estimating DSS. The secondary endpoint was overall survival (OS), which was defined as the time from diagnosis to death or last follow-up.

### Statistical analyses

Patient characteristics were presented as mean ± standard deviation for continuous variables, and number with percentage for categorical variables. The differences of clinopathological characteristics between patients received RAI and those who did not were assessed using Mann–Whitney U test, chi-square or Fisher exact test according to the variable distribution. DSS and OS differences were analyzed by Kaplan–Meier method and the log-rank test. The variables associated independently with DSS or OS were identified by the Cox proportional hazards models with adjusted hazard ratios (HRs) and 95% confidence intervals (CIs). To further assess the prognostic impact of RAI ablation, subgroup analyses were performed based on clinicopathological characteristics. All *P* values are two-sided and *P*<0.05 is considered statistically significant. All analyses were performed using the SPSS ver. 22.0 (IBM Corp., Armonk, NY, USA).

## Results

### Clinicopathological characteristics

A total of 23107 intermediate-risk PTC patients were enrolled in our study. Of those, 16212 (70.2%) received RAI ablation and 6895 (29.8%) did not. There were significant differences in demographic and clinicopathological features between patients who received RAI and those who did not. Young age, multifocality, extrathyroidal extension and regional lymph node metastasis were associated with an increased likelihood of receiving RAI ablation. (Table 1).

**Table 1:** Clinicopathological characteristics of intermediate risk PTC patients with or without RAI ablation. (N = 23107).

Variables	No-RAI N = 6895 (29.8%)	RAI N = 16212 (70.2%)	*P* value
Race			< 0.001
White	5466 (79.3)	13086 (80.7)	
Black	448 (6.5)	674 (4.2)	
other	981 (14.2)	2452 (15.1)	
Gender			0.001
Female	4870 (70.6)	11791 (72.7)	
Male	2025 (29.4)	4421 (27.3)	
Age			
Mean±SD (years)	46.5±16.14	44.1 ± 14.82	< 0.001
< 45 years	3283 (47.6)	8470 (52.3)	
45–55 years	1458 (21.1)	3756 (23.2)	
55–65 years	1155 (16.8)	2406 (14.8)	
≥65 years	999 (14.5)	1580 (9.7)	< 0.001
Multifocality	3175(46.0)	8760 (54.0)	< 0.001
Tumor size			
Mean±SD (mm)	29.0 ± 27.21	25.3 ± 18.58	< 0.001
≤10 mm	1332 (19.3)	3072 (18.9)	
10–20 mm	2073 (30.1)	5529 (34.1)	
20–40 mm	1789 (25.9)	4526 (27.9)	
> 40 mm	1701 (24.7)	3085 (19.1)	< 0.001
Regional LN metastasis			< 0.001
Absent	1091 (15.8)	2164 (13.5)	
Present	4172 (60.5)	11355 (70.0)	< 0.001
CLNM	2477 (59.4)	6879 (60.6)	
LLNM	1094 (26.2)	3305 (29.1)	
NOS	601 (14.4)	1171 (10.3)	
Not examined	1632 (23.7)	2676 (16.5)	
ETE	2838 (41.2)	7201 (44.4)	< 0.001
Vital status			
Alive	6540 (94.9)	15958 (98.4)	< 0.001
Overall death	355 (5.1)	254 (1.6)	< 0.001
Cancer-specific death	163 (2.4)	82 (0.5)	< 0.001
Survival			
10-year DSS	95.7%	98.3%	<0.001
10-year OS	88.7%	95.4%	<0.001
Median follow-up (months) (IQR)	44 (19–79)	48 (22–80)	< 0.001

PTC, papillary thyroid cancer; RAI, radioactive iodine; SD, standard deviation; LN, lymph node; CLNM, central lymph node metastasis; LLNM, lateral lymph node metastasis; NOS, not otherwise specified; ETE, extrathyroidal extension; DSS, disease-specific survival; OS, overall survival; IQR, interquartile range.

### Prognostic impact of RAI ablation on DSS and OS

**Fig 1:**
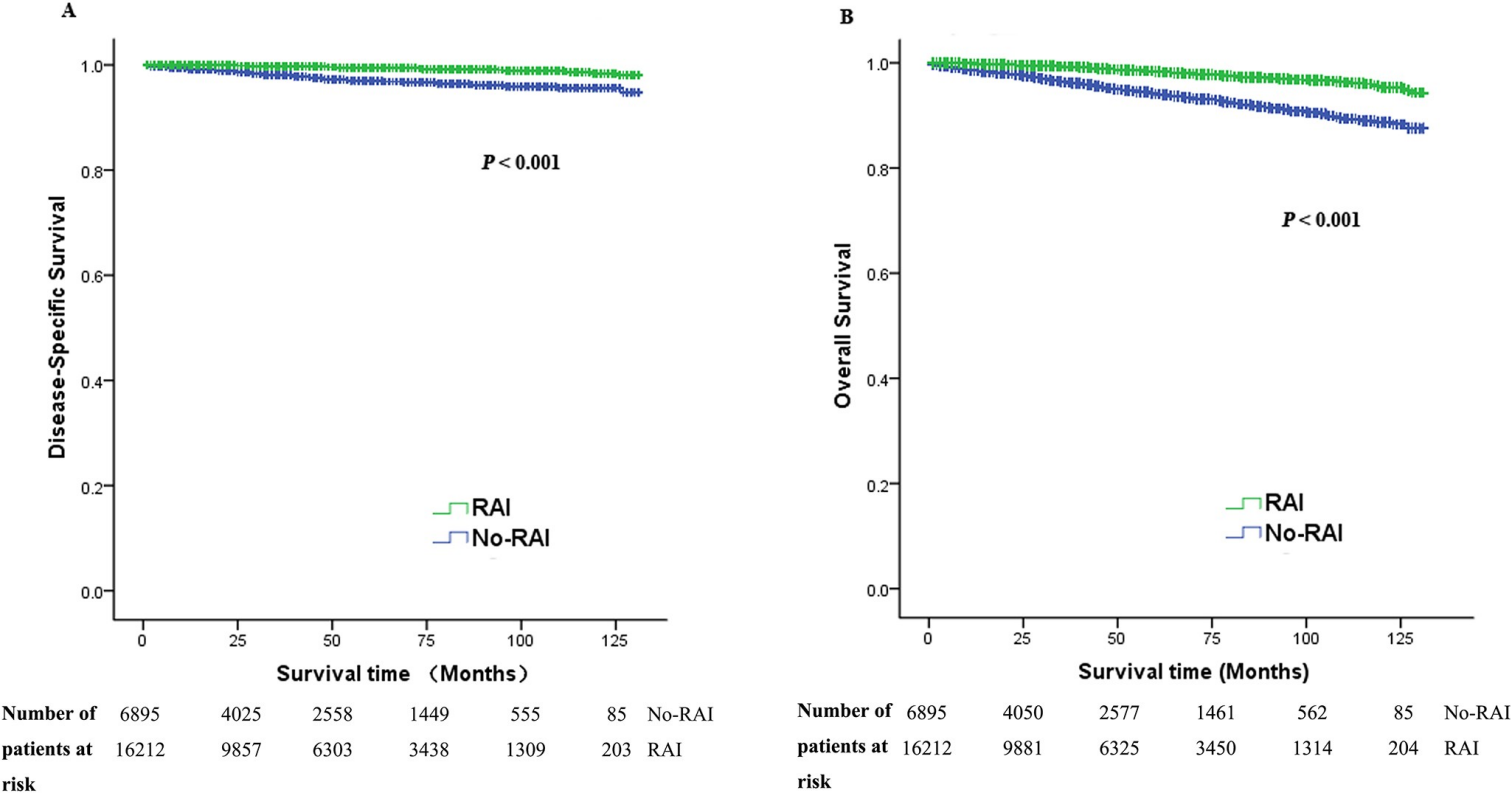
Unadjusted disease-specific survival (A) and overall survival (B) difference for intermediate-risk PTC patients according to RAI therapy.

The median follow-up time was 47 months (range: 6–131 months, IQR: 21–80 months) for all patients. The median follow-up time of no-RAI group was significantly shorter than that of the RAI ablation group (44 vs 48 months, *P* < 0.001). During the follow-up period, 609 deaths (2.6%) were observed, of which 245 deaths (1.1%) were due to PTC-specific death. Details of death were presented in Table 1. The DSS and OS were significantly higher for patients received RAI than those who did not (log-rank, both *P* < 0.001) (Table 1 and Fig 1).

After adjusting for clinicopathological characteristics using the Cox proportional hazards model (Table 2), RAI therapy was significantly associated with reduced disease-specific death (adjusted HR = 0.65, *P* = 0.017) and overall death (adjusted HR = 0.54, *P* < 0.001) in intermediate-risk PTC patients. Male gender, older age (≥ 45 years), larger tumor (> 20 mm), multifocality, extrathyroidal extension and lymph node metastases significantly increased the risk of disease-specific death or overall death when controlling for the remaining variables (all *P* < 0.05).

**Table 2:** Cox proportional hazards model for disease-specific death or overall death in intermediate risk PTC patients.

	Disease-specific death		Overall death	
	Adjusted HR (95% CI)	*P* value	Adjusted HR (95% CI)	*P* value
Male gender	1.50 (1.07–2.10)	0.019	1.60 (1.32–1.93)	< 0.001
Age at diagnosis (years)				
< 45	1		1	
45–55	8.23 (3.51–19.32)	< 0.001	3.80 (2.57–5.63)	< 0.001
55–65	21.38 (9.51–48.06)	< 0.001	9.07 (6.27–13.11)	< 0.001
≥65	55.11 (25.10–121.03)	< 0.001	34.60 (24.68–48.52)	< 0.001
Extent of surgery (TT)	0.29 (0.16–0.54)	< 0.001	0.56 (0.36–0.89)	0.014
Tumor size (mm)				
≤10	1		1	
10–20	1.05 (0.48–2.28)	0.906	1.15 (0.84–1.58)	0.393
20–40	2.57 (1.28–5.16)	0.008	1.40 (1.02–1.92)	0.036
> 40	6.50 (3.31–12.78)	< 0.001	2.24 (1.63–3.07)	< 0.001
Regional LN metastasis				
Absent	1		1	
Present	2.83 (1.67–4.82)	< 0.001	2.00 (1.48–2.72)	< 0.001
Not examined	0.92 (0.51–1.66)	0.787	1.17 (0.85–1.60)	0.333
Multifocality	2.03 (1.40–2.95)	< 0.001	1.26 (1.04–1.52)	0.021
ETE (+)	1.91 (1.36–2.69)	< 0.001	1.38 (1.13–1.68)	0.002
RAI (+)	0.65 (0.46–0.93)	0.017	0.54 (0.44–0.65)	< 0.001

PTC, papillary thyroid cancer; HR, hazard ratios; CI, confidence interval; ETE, extrathyroidal extension; RAI, radioactive iodine.

Subgroup analyses (Table 3) demonstrated that RAI ablation was only associated with improved DSS in patients with male gender (adjusted HR = 0.47, *P* = 0.005), age ≥ 45 years (adjusted HR = 0.34, *P* < 0.001) and tumor size > 20 mm (adjusted HR = 0.58, *P* = 0.007), but this association was not observed in other subgroups, even in patients with several aggressive features such as multifocality (adjusted HR = 0.70, *P* = 0.276), ETE (adjusted HR = 0.68, *P* = 0.108) and lymph node metastasis (adjusted HR = 0.70, *P* = 0.128). However, RAI had significantly influence on OS based on different clinicopathological features in all subgroup analyses, except patients with age < 45 years, underwent NTT/STT, and no lymph node metastasis.

**Table 3:** Subgroup analyses of the impact of RAI on DSS or OS in intermediate risk PTC patients.

	DSS^#^		OS^#^	
	Adjusted HR of RAI (95% CI)	*P* value	Adjusted HR of RAI (95% CI)	*P* value
Gender				
Male	0.47 (0.28–0.80)	0.005*	0.46 (0.35–0.62)	< 0.001*
Female	0.91 (0.56–1.49)	0.703	0.59 (0.46–0.76)	< 0.001*
Age at diagnosis				
< 45 years	0.51 (0.14–1.86)	0.305	0.60 (0.31–1.13)	0.113
≥ 45 years	0.34 (0.25–0.46)	<0.001*	0.51 (0.42–0.63)	< 0.001*
Tumor size				
≤ 20 mm	0.61 (0.28–1.30)	0.197	0.60 (0.44–0.82)	0.001*
> 20 mm	0.58 (0.39–0.86)	0.007*	0.47 (0.37–0.59)	<0.001*
Multifocality				
Absent	0.68 (0.44–1.05)	0.082	0.56 (0.43–0.72)	< 0.001*
Present	0.70 (0.37–1.33)	0.276	0.52 (0.39–0.69)	< 0.001*
Regional LN metastasis				
Absent	1.05 (0.38–2.90)	0.926	0.57 (0.21–1.58)	0.203
Present	0.70 (0.44–1.11)	0.128	0.49 (0.38–0.63)	< 0.001*
CLNM	1.11 (0.50–2.44)	0.801	0.51 (0.35–0.71)	< 0.001*
LLNM	0.55 (0.25–1.19)	0.127	0.50 (0.31–0.80)	0.004*
ETE				
Absent	0.63 (0.36–1.12)	0.112	0.42 (0.32–0.56)	< 0.001*
Present	0.68 (0.43–1.09)	0.108	0.66 (0.51–0.87)	0.003*

RAI, radioactive iodine; DSS, disease-specific survival; OS, overall survival; PTC, papillary thyroid cancer; HR, hazard ratios; CI, confidence interval; LN, lymph node; CLNM, central lymph node metastasis; LLNM, lateral lymph node metastasis; ETE, extrathyroidal extension.

^#^ Adjusted for gender, age at diagnosis, tumor size, multifocality, regional LN metastasis and ETE, except for the subgroup variable being studied.

## Discussion

The concept of risk stratification is critical for understanding the biological behavior of the disease and planning treatment. According to their relative risk of recurrence and death, thyroid cancer patients were divided into high, intermediate, and low-risk groups by the ATA guidelines [18]. High-risk patients may have gross extrathyroidal extension, incomplete tumor resection or distant metastases. Intermediate-risk patients have microscopic extrathyroidal extension, regional lymph node metastasis, vascular invasion, or aggressive histology (e.g. tall cell, hobnail variant, columnar cell carcinoma). Low-risk patients have no extrathyroidal extension, regional or distant metastasis, vascular invasion, or aggressive histology. Because the risk of disease-specific mortality and persistent/recurrent disease is low and RAI treatment has little benefit for low-risk patients [19], RAI adjuvant therapy is not routinely recommended for low-risk PTC patients after total thyroidectomy. There are ample studies confirmed that RAI can reduce the risk of recurrence and improve the overall survival for high-risk PTC patients, while the benefit of RAI for intermediate-risk patients was unclear. Under these evidences, RAI treatment is routinely recommended for high-risk PTC patients with strong recommendation, while RAI treatment should be considered after total thyroidectomy in intermediate-risk patients with weak recommendation because of low-quality evidence according to the ATA guidelines [18].

Until now, only a few retrospective studies have specifically studied the effect of RAI treatment in intermediate-risk group, but these findings were controversial. Multivariate adjusted analyses from NCDB showed that RAI treatment can significantly improve overall survival in intermediate-risk PTC patients [16]. In a retrospective study, Chow SM et al [20] analyzed the subgroup of 352 patients with microscopic extrathyroidal extension and found that RAI treatment can significantly reduce the risk of recurrence. However, in a single institution retrospective studies from South Korea analyzing data from a cohort of 8297 intermediate-risk PTC patients, RAI treatment was not significantly related to the risk of loco-regional recurrence after adjusting for clinicopathological characteristics [17]. Another study from Korea also indicated that RAI treatment did not decrease recurrence in intermediate-risk patients with papillary thyroid microcarcinoma [21]. A recent systematic review reported conflicting results on the effect of RAI therapy on disease recurrence in intermediate-risk patients. The authors found that 11 original studies observed some benefit in reducing recurrence with RAI treatment, while 13 studies failed to show benefit [22]. Several hypotheses can be proposed to explain these inconsistent results. First, due to ethnic differences, there may be different prognostic factors. Second, because of the inertness of PTC, in some studies, the follow-up period might be too short to detect a significant difference in recurrence or survival. Third, different centers have different treatment protocols, such as dealing with lymph nodes of central neck area, some are routine prophylactic CND, and some are not, which might have an important impact on RAI for recurrence or survival.

In our study, we conducted a retrospective large-sample cohort analysis based on SEER database and found that RAI can increased the OS in majority of patients with intermediate-risk PTC, except patients with age < 45 years, underwent NTT/STT, and no lymph node metastasis. This finding differed from the previous NCDB study which suggested that RAI was associated with an increased overall survival in all patients including patients < 45 years [16]. Unlike the NCDB, the SEER database also provides disease-specific death information, therefore we could analyze DSS in addition to OS. Our results indicated that RAI can increased the DSS in all patients (adjusted HR = 0.65, *P* = 0.017). To make the results more homogeneous, subgroup analyses were conducted stratified by clinicopathological features, and we found that the benefits of RAI treatment were only observed in patients with male gender (adjusted HR = 0.47, *P* = 0.005), age ≥ 45 years (adjusted HR = 0.57, *P* = 0.002) and tumor size > 20 mm (adjusted HR = 0.58, *P* = 0.007). However, DSS was not affected by the use of RAI regardless of ETE and lymph node metastasis, even in patients with lateral lymph node metastasis (LLNM). ETE is divided into microscopic ETE and gross ETE. Only microscopic ETE belongs to the intermediate-risk group, gross ETE belongs to the high-risk group. It is not difficult to understand that the ETE has no impact on RAI ablation for DSS in intermediate-risk PTC patients, because many recent studies have shown that microscopic ETE is not related to recurrence-free survival [23,24]. However, it seems to contradict the clinical impression regarding the effect of lymph node metastasis, especially lateral lymph node metastasis on RAI ablation for DSS. Clinically lymph nodes metastasis is associated with recurrence [25,26], but the effect on survival is controversial. Following the current guidelines, clinically suspicious LLNM should be removed by therapeutic lateral neck dissection (LND). Similar to the group without LND, the LND group had little chance to detect undetected LLNM after LND. Therefore, their prognosis may be similar in patients with or without LLNM.

Age and tumor size are important prognostic factors for PTC and have been used as major stratification factors in prominent classification systems such as TNM (Tumor, Nodes, Metastases), MACIS (Metastases, Age, Completeness of resection, Invasion, Size) and AMES (Age, Metastases, Extent, Size) [27–29]. Old age and larger tumor size were associated with higher recurrence and death risk in patients with PTC. Therefore, a more aggressive treatment including RAI therapy for these patients has been advocated by some researchers [30,31]. Whether male gender is a negative prognostic factor for PTC is controversial. Some studies supported this correlation between male gender and adverse outcome, but other studies failed to show the prognostic value of gender, which hypothesized that worse outcomes in men may potentially be accounted for by a more aggressive behavior of PTC in these patients [32]. Whether the aforementioned factors affect the efficacy of RAI is not conclusive. Our study based on large-sample data indicated that RAI ablation only benefits intermediate-risk PTC patients with male gender, age ≥ 45 years and tumor size > 20 mm.

Our study has several limitations. There may be coding errors in the SEER database. Although SEER performed annual audits, no centralized review of pathologic specimens by thyroid pathologist is performed. However, the SEER was highly standardized and its accuracy has been described in numerous studies [33,34]. In addition, the SEER database didn’t record the information related to patient comorbidities, vascular invasion, recurrence, biochemical data (such as thyroglobulin, thyroid stimulating hormone, and thyroxine levels), the ablation doses and frequency and pertinent molecular markers (such as BRAF, RET and TERT gene status), which may affect the clinical response to RAI therapy for patients with PTC.

## Conclusion

This is the first nationally representative study to address the association of RAI therapy with DSS for intermediate-risk PTC patients, as defined by ATA and AJCC criteria. RAI decision-making should be considered on an individual basis rather than “one size fits all” in intermediate-risk PTC patients; only patients with male gender, age ≥ 45 years and tumor size > 20 mm may benefit from RAI therapy. However, due to the potential limitations, further more studies including randomized controlled trial are needed to confirm or adjust our findings.

## References

[1] Davies L, Welch HG (2006) Increasing incidence of thyroid cancer in the United States, 1973–2002. JAMA 295: 2164–2167. 16684987 10.1001/jama.295.18.2164

[2] Aschebrook-Kilfoy B, Ward MH, Sabra MM, Devesa SS (2011) Thyroid cancer incidence patterns in the United States by histologic type, 1992–2006. Thyroid 21: 125–134. 10.1089/thy.2010.0021 21186939 PMC3025182

[3] Carhill AA, Litofsky DR, Ross DS, Jonklaas J, Cooper DS, et al (2015) Long-Term Outcomes Following Therapy in Differentiated Thyroid Carcinoma: NTCTCS Registry Analysis 1987–2012. J Clin Endocrinol Metab 100: 3270–3279. 10.1210/JC.2015-1346 26171797 PMC5393522

[4] Liu FH, Kuo SF, Hsueh C, Chao TC, Lin JD (2015) Postoperative recurrence of papillary thyroid carcinoma with lymph node metastasis. J Surg Oncol 112: 149–154. 10.1002/jso.23967 26175314 PMC5034820

[5] Brierley J, Tsang R, Panzarella T, Bana N (2005) Prognostic factors and the effect of treatment with radioactive iodine and external beam radiation on patients with differentiated thyroid cancer seen at a single institution over 40 years. Clin Endocrinol (Oxf) 63: 418–427. 16181234 10.1111/j.1365-2265.2005.02358.x

[6] Mazzaferri EL, Jhiang SM (1994) Long-term impact of initial surgical and medical therapy on papillary and follicular thyroid cancer. Am J Med 97: 418–428. 10.1016/0002-9343(94)90321-2 7977430

[7] Tsang RW, Brierley JD, Simpson WJ, Panzarella T, Gospodarowicz MK, et al (1998) The effects of surgery, radioiodine, and external radiation therapy on the clinical outcome of patients with differentiated thyroid carcinoma. Cancer 82: 375–388. 9445196

[8] Haymart MR, Banerjee M, Stewart AK, Koenig RJ, Birkmeyer JD, et al (2011) Use of radioactive iodine for thyroid cancer. JAMA 306: 721–728. 10.1001/jama.2011.1139 21846853 PMC3352591

[9] Iyer NG, Morris LG, Tuttle RM, Shaha AR, Ganly I (2011) Rising incidence of second cancers in patients with low-risk (T1N0) thyroid cancer who receive radioactive iodine therapy. Cancer 117: 4439–4446. 10.1002/cncr.26070 21432843 PMC3155861

[10] Kloos RT, Duvuuri V, Jhiang SM, Cahill KV, Foster JA, et al (2002) Nasolacrimal drainage system obstruction from radioactive iodine therapy for thyroid carcinoma. J Clin Endocrinol Metab 87: 5817–5820. 10.1210/jc.2002-020210 12466391

[11] Klubo-Gwiezdzinska J, Van Nostrand D, Burman KD, Vasko V, Chia S, et al (2010) Salivary gland malignancy and radioiodine therapy for thyroid cancer. Thyroid 20: 647–651. 10.1089/thy.2009.0466 20470209

[12] Lee SL (2010) Complications of radioactive iodine treatment of thyroid carcinoma. J Natl Compr Canc Netw 8: 1277–1286; quiz 1287. 10.6004/jnccn.2010.0094 21081784

[13] Buffet C, Golmard JL, Hoang C, Tresallet C, Du Pasquier Fediaevsky L, et al (2012) Scoring system for predicting recurrences in patients with papillary thyroid microcarcinoma. Eur J Endocrinol 167: 267–275. 10.1530/EJE-12-0105 22648965

[14] Creach KM, Siegel BA, Nussenbaum B, Grigsby PW (2012) Radioactive iodine therapy decreases recurrence in thyroid papillary microcarcinoma. ISRN Endocrinol 2012: 816386 10.5402/2012/816386 22462017 PMC3313572

[15] Lee J, Song Y, Soh EY (2014) Prognostic significance of the number of metastatic lymph nodes to stratify the risk of recurrence. World J Surg 38: 858–862. 10.1007/s00268-013-2345-6 24305921

[16] Ruel E, Thomas S, Dinan M, Perkins JM, Roman SA, et al (2015) Adjuvant radioactive iodine therapy is associated with improved survival for patients with intermediate-risk papillary thyroid cancer. J Clin Endocrinol Metab 100: 1529–1536. 10.1210/jc.2014-4332 25642591 PMC4399282

[17] Kim SK, Woo JW, Lee JH, Park I, Choe JH, et al (2016) Radioactive iodine ablation may not decrease the risk of recurrence in intermediate-risk papillary thyroid carcinoma. Endocr Relat Cancer 23: 367–376. 10.1530/ERC-15-0572 26917553

[18] Haugen BR, Alexander EK, Bible KC, Doherty GM, Mandel SJ, et al (2016) 2015 American Thyroid Association Management Guidelines for Adult Patients with Thyroid Nodules and Differentiated Thyroid Cancer: The American Thyroid Association Guidelines Task Force on Thyroid Nodules and Differentiated Thyroid Cancer. Thyroid 26: 1–133. 10.1089/thy.2015.0020 26462967 PMC4739132

[19] Sacks W, Fung CH, Chang JT, Waxman A, Braunstein GD (2010) The effectiveness of radioactive iodine for treatment of low-risk thyroid cancer: a systematic analysis of the peer-reviewed literature from 1966 to April 2008. Thyroid 20: 1235–1245. 10.1089/thy.2009.0455 21062195

[20] Chow SM, Yau S, Kwan CK, Poon PC, Law SC (2006) Local and regional control in patients with papillary thyroid carcinoma: specific indications of external radiotherapy and radioactive iodine according to T and N categories in AJCC 6th edition. Endocr Relat Cancer 13: 1159–1172. 10.1677/erc.1.01320 17158761

[21] Kim HJ, Kim NK, Choi JH, Kim SW, Jin SM, et al (2013) Radioactive iodine ablation does not prevent recurrences in patients with papillary thyroid microcarcinoma. Clin Endocrinol (Oxf) 78: 614–620. 22957654 10.1111/cen.12034

[22] Lamartina L, Durante C, Filetti S, Cooper DS (2015) Low-risk differentiated thyroid cancer and radioiodine remnant ablation: a systematic review of the literature. J Clin Endocrinol Metab 100: 1748–1761. 10.1210/jc.2014-3882 25679996

[23] Moon HJ, Kim EK, Chung WY, Yoon JH, Kwak JY (2011) Minimal extrathyroidal extension in patients with papillary thyroid microcarcinoma: is it a real prognostic factor? Ann Surg Oncol 18: 1916–1923. 10.1245/s10434-011-1556-z 21267788

[24] Woo CG, Sung CO, Choi YM, Kim WG, Kim TY, et al (2015) Clinicopathological Significance of Minimal Extrathyroid Extension in Solitary Papillary Thyroid Carcinomas. Ann Surg Oncol 22 Suppl 3: S728–733. 26077913 10.1245/s10434-015-4659-0 PMC4686556

[25] Pitoia F, Bueno F, Urciuoli C, Abelleira E, Cross G, et al (2013) Outcomes of patients with differentiated thyroid cancer risk-stratified according to the American thyroid association and Latin American thyroid society risk of recurrence classification systems. Thyroid 23: 1401–1407. 10.1089/thy.2013.0011 23517313

[26] Tuttle RM, Tala H, Shah J, Leboeuf R, Ghossein R, et al (2010) Estimating risk of recurrence in differentiated thyroid cancer after total thyroidectomy and radioactive iodine remnant ablation: using response to therapy variables to modify the initial risk estimates predicted by the new American Thyroid Association staging system. Thyroid 20: 1341–1349. 10.1089/thy.2010.0178 21034228 PMC4845674

[27] Amin MB E S, Greene FL, et al (2016) AJCC Cancer StagingManual (ed 8). NewYork, NY, Springer.

[28] Cady B, Rossi R (1988) An expanded view of risk-group definition in differentiated thyroid carcinoma. Surgery 104: 947–953. 3194846

[29] Hay ID, Bergstralh EJ, Goellner JR, Ebersold JR, Grant CS (1993) Predicting outcome in papillary thyroid carcinoma: development of a reliable prognostic scoring system in a cohort of 1779 patients surgically treated at one institution during 1940 through 1989. Surgery 114: 1050–1057; discussion 1057–1058. 8256208

[30] Ito Y, Miyauchi A, Kihara M, Takamura Y, Kobayashi K, et al (2012) Relationship between prognosis of papillary thyroid carcinoma patient and age: a retrospective single-institution study. Endocr J 59: 399–405. 10.1507/endocrj.ej12-0044 22374240

[31] Sugino K, Kure Y, Iwasaki H, Ozaki O, Mimura T, et al (1995) Metastases to the regional lymph nodes, lymph node recurrence, and distant metastases in nonadvanced papillary thyroid carcinoma. Surg Today 25: 324–328. 10.1007/BF00311254 7633123

[32] Glikson E, Alon E, Bedrin L, Talmi YP (2017) Prognostic Factors in Differentiated Thyroid Cancer Revisited. Isr Med Assoc J 19: 114–118. 28457063

[33] Hankey BF, Ries LA, Edwards BK (1999) The surveillance, epidemiology, and end results program: a national resource. Cancer Epidemiol Biomarkers Prev 8: 1117–1121. 10613347

[34] Harlan LC, Hankey BF (2003) The surveillance, epidemiology, and end-results program database as a resource for conducting descriptive epidemiologic and clinical studies. J Clin Oncol 21: 2232–2233. 10.1200/JCO.2003.94.023 12805320

